# Imaging as a Surgical Decision System in Modern Gynecologic Oncology

**DOI:** 10.3390/diagnostics16213666

**Published:** 2026-11-06

**Authors:** Angel Yordanov, Vasilena Dimitrova Dimitrova, Stoyan Kostov, Ihsan Hasan, Samar El Shemeri, Eva Tsoneva

**Affiliations:** 1 Department of Gynaecological Oncology, Medical University Pleven, 5800 Pleven, Bulgaria; vasilenadimitrova4@gmail.com; 2 Research Institute, Medical University Pleven, 5800 Pleven, Bulgaria; drstoqn.kostov@gmail.com; 3 Department of Gynecology, St. Anna University Hospital, 9002 Varna, Bulgaria; 4 Department of Gynaecology and Pelvic Surgery, Hospital (MHAT) Vita 2, 1407 Sofia, Bulgaria; ihsan_hasanov@abv.bg; 5 Department of Medical Imaging, Heart and Brain Center of Excellence, 8000 Burgas, Bulgaria; s.shemeri.bs@heartandbrain.bg; 6 Gynecology Clinic, Military Medical Academy, 1606 Sofia, Bulgaria; dretsoneva@gmail.com

**Keywords:** gynecologic oncology, medical imaging, surgical decision-making, imaging-target mismatch, surgical window, oligoprogression, intraoperative imaging, radiomics, artificial intelligence

## Abstract

Imaging traditionally supports gynecologic oncology through diagnosis, staging, and estimation of resectability. Contemporary treatment, however, requires longitudinal decisions about which lesion matters, when local intervention is justified, and which modality is proportionate. This critical narrative review proposes imaging as a longitudinal surgical decision system integrating disease trajectory, treatment response, target selection, operative planning, and post-intervention assessment. We define imaging-target mismatch as discordance between the radiologically dominant lesion and the lesion with greatest clinical actionability, and operationalize the disease-modifying surgical window as open, uncertain, or closed across six decision domains. Serial imaging may reveal response-created opportunities, oligopersistence, oligoprogression, competing dissemination, or the need for tissue confirmation, while CT, MRI, ultrasound, and FDG PET/CT provide complementary decision-specific information. Three-dimensional reconstruction, intraoperative imaging, radiomics, and artificial intelligence may enhance planning, but require prospective external validation and should support rather than replace multidisciplinary judgment. Decision-oriented reporting should communicate the target, lesion-level trajectory, competing disease, anatomical feasibility, residual uncertainty, and the relevance of findings to the proposed intervention. Imaging should therefore guide not only what can be treated, but what should be treated, when, and for what therapeutic purpose.

## 1. Introduction

Imaging has traditionally supported gynecologic oncology surgery by defining disease extent, assigning stage, and estimating technical resectability. That role remains fundamental but does not capture its full contribution to contemporary decision-making. Systemic therapy can alter disease distribution, create opportunities for localized intervention, or reveal spatially restricted resistance. Both resectability and the potential value of surgery may therefore change during treatment rather than remain fixed at diagnosis [1].

The relevant question is no longer only where disease is located, but which lesion matters, when it should be targeted, and what local treatment is intended to achieve. A residual post-treatment mass may contain viable tumor, fibrosis, or both, whereas progression at one site during otherwise effective systemic therapy may identify a clinically important focus of resistance. Conversely, apparently limited disease may coexist with occult dissemination that substantially reduces the expected value of surgery. Imaging therefore contributes to target selection, timing, and definition of the therapeutic objective as well as anatomical mapping.

We propose that imaging should be considered a longitudinal surgical decision system in gynecologic oncology. Serial imaging maps disease trajectory, identifies or excludes a meaningful target, informs comparison of surgery with other local treatments, guides operative planning, and evaluates whether the intended objective was achieved. Imaging should not determine surgical candidacy in isolation. Findings must be integrated with treatment response, tumor biology, patient fitness, expected morbidity, and the feasibility of less invasive alternatives [2,3,4]. The practical aim is to translate imaging findings into decision-oriented reporting and multidisciplinary communication.

Accordingly, this article is conceived as a critical narrative review and conceptual framework rather than a hypothesis-testing study. Its purpose is to integrate available evidence and multidisciplinary practice into a decision-oriented model for imaging beyond diagnosis and staging, and to define clinically testable constructs and research priorities for prospective validation.

Evidence informing this conceptual framework was identified through targeted searches of PubMed/MEDLINE, Scopus, and Web of Science through October 2026. Search concepts included gynecologic cancer, surgery, cytoreduction, salvage surgery, local therapy, medical imaging, resectability, treatment response, oligoprogression, radiomics, and artificial intelligence. Priority was given to clinical guidelines, consensus statements, randomized and prospective studies, and imaging investigations directly relevant to surgical decision-making. Reference lists of key publications were also reviewed. Because the objective was concept development rather than comprehensive evidence synthesis or effect estimation, study selection was purposive and iterative, and no formal risk-of-bias assessment was performed.

## 2. Beyond Staging

Staging provides an anatomical snapshot at a defined time point, whereas surgical decisions increasingly depend on disease trajectory across treatment. The same distribution may have different implications before systemic therapy, after a major response, during oligopersistence, or at oligoprogression. Surgical relevance therefore depends on temporal behavior and clinical context as well as location and size.

When imaging is used to assess operability, it can identify disease at surgically critical sites, anticipate multidisciplinary procedures, and show patterns that make complete cytoreduction unlikely. However, all modalities can underestimate small-volume, diffuse, serosal, or mesenteric disease. Current consensus recommendations support imaging for operability assessment and treatment planning but caution against using it as the sole determinant of peritoneal resectability [2,3,4].

A further limitation is that conventional response assessment is primarily lesion- and size-based. Such measurements provide reproducible evidence of tumor change but do not necessarily identify the lesion that is biologically resistant, surgically actionable, or most likely to alter subsequent management [5]. A small enlarging lesion during otherwise effective treatment may be more relevant to the surgical decision than a larger but responding mass. Similarly, radiological stability does not establish that residual disease is biologically inactive or that its removal would improve outcome.

Beyond staging, interpretation should begin with the clinical decision: whether a meaningful target exists, whether local treatment could alter the therapeutic course, and whether the proposed intervention is proportionate. Three-dimensional segmentation and patient-specific reconstruction may improve translation of cross-sectional imaging into an operative plan, but current evidence is limited. The recent prospective proof-of-concept study included only four patients and demonstrates feasibility, not clinical benefit [6].

In this model, imaging does not simply document what can be removed. It helps determine what should be targeted, when intervention is justified, and whether the disease-modifying surgical window is open, uncertain, or closed.

## 3. Imaging Defines the Surgical Target

Detecting a lesion does not by itself define a surgical target. A visible and technically resectable abnormality may have little therapeutic relevance, whereas a smaller or less accessible lesion may drive resistance or the next treatment decision. Imaging should therefore progress from lesion detection to target qualification: determining whether a particular site is clinically meaningful, sufficiently isolated, and actionable by local treatment.

A qualified target must first be anatomically defined. Imaging should describe its relationship to vessels, bowel, urinary tract, nerves, bone, and previously treated tissues, and estimate whether complete local control is achievable with acceptable morbidity. It must also be assessed in the context of the total disease burden. A single visible lesion is not truly isolated when occult peritoneal, nodal, or distant disease is likely. Because imaging may miss diffuse or subcentimeter disease, apparent isolation should be interpreted cautiously, particularly before major surgery [2,3,4].

The target must also be therapeutically relevant. In oligoprogression, one or a few lesions progress while the remaining visible disease appears controlled during systemic therapy. This pattern may reflect spatially restricted treatment resistance, but imaging cannot establish that the current regimen remains biologically effective or that occult resistant disease is absent. Imaging identifies this pattern through the number, distribution, and lesion-level trajectory of visible lesions [7]. Local treatment may control the progressing focus and permit continuation of the current regimen in selected patients; however, this should be regarded as a treatment rationale rather than an established survival-improving strategy.

Gynecologic cancer-specific evidence remains limited and observational. A multi-institutional cohort of 215 patients treated with stereotactic ablative radiotherapy for gynecologic cancers, including 86 with oligoprogression, reported favorable local control. Median chemotherapy-free survival was 21.7 months among the 178 patients who did not receive immediate chemotherapy after radiotherapy; this estimate was not specific to oligoprogression [8]. In a 30-patient ovarian cancer series, surgery or stereotactic radiotherapy was used for progression during PARP-inhibitor maintenance, with median prolongation of the platinum-free interval of 6 and 10 months, respectively [9]. A separate 34-patient case series during immune-checkpoint blockade included surgery, ablation, and radiotherapy and reported feasibility, with a median progression-free survival of 5.3 months after intervention [10]. Because these studies were retrospective, uncontrolled, heterogeneous, and highly selected, they demonstrate feasibility and local disease control but do not establish that local treatment prolongs survival or that continuing systemic therapy is superior to switching treatment. Preservation of the current regimen should therefore be framed as a clinically plausible, selection-dependent strategy rather than a demonstrated survival benefit.

The most obvious lesion is not necessarily the appropriate target. The largest lesion may be responding, the most metabolically active lesion may be unsafe to resect, and the most accessible lesion may yield tissue without contributing to disease control. We use imaging-target mismatch to describe discordance between radiological dominance and clinical actionability. Visibility, measurability, accessibility, biological informativeness, and therapeutic relevance may converge in one site, but should not be assumed to do so.

This distinction is particularly important when surgery is considered for both local control and tissue acquisition. A lesion that behaves differently from the remaining disease may contain resistant tumor, but imaging cannot establish the molecular mechanism. It should guide selection of the lesion most likely to yield management-changing tissue and help determine whether image-guided biopsy, limited resection, or another local treatment is most appropriate. Ultrasound-guided core-needle biopsy can provide adequate diagnostic tissue in selected patients with gynecologic malignancy and should be considered when excision would add morbidity without a separate therapeutic objective [11].

Target definition should therefore precede modality selection. The multidisciplinary team should first clarify what the intervention is expected to achieve: durable local control, eradication of a resistant focus, prevention of clinically consequential progression, symptom relief, or acquisition of tissue that could change treatment. Only then should surgery be compared with stereotactic radiotherapy, image-guided ablation, or biopsy. Imaging supports this comparison by defining target size, location, accessibility, proximity to critical structures, and the presence of competing disease elsewhere [12].

Accordingly, an imaging-defined surgical target is not simply a lesion that can be removed. It is a lesion whose local treatment has a plausible capacity to alter the patient’s therapeutic trajectory and for which surgery may offer a context-specific and clinically plausible advantage over observation, systemic treatment, biopsy, or another local modality. This definition places imaging at the beginning of surgical reasoning: not as proof that an operation is indicated, but as the basis for deciding whether a coherent surgical target exists at all.

## 4. Imaging Across the Treatment Trajectory

Pretreatment imaging maps baseline disease distribution and identifies anatomical factors that may affect surgery. After systemic treatment, the question shifts from initial extent to response pattern: global response, spatially limited persistence, widespread progression, or progression at one or a few sites [2].

Serial comparison is therefore more informative than a single examination. It may reveal a new opportunity for surgery after major response, persistent macroscopic disease suitable for consolidation, or oligoprogression during otherwise effective therapy. Conversely, new lesions, previously occult dissemination, or rapidly evolving multifocal disease may close the disease-modifying surgical window even when one lesion remains technically resectable.

Imaging influences management beyond staging when it changes the timing, modality, or extent of local treatment. After systemic therapy, interval imaging may show that previously diffuse disease has evolved into a limited and anatomically controllable target, or conversely reveal new mesenteric, serosal, or distant disease that removes the rationale for surgery [4]. In oligoprogression, serial imaging may identify one or a few escaping lesions while the remaining visible disease appears controlled. This pattern may support multidisciplinary consideration of local treatment and continuation of the current systemic regimen, but the available gynecologic evidence is observational and does not demonstrate that this strategy improves survival [7,8,9,10]. In suspected central pelvic recurrence, MRI, CT, and FDG PET/CT may determine whether disease remains confined to a surgically controllable compartment, whether pelvic sidewall or distant involvement makes exenterative surgery disproportionate, and whether image-guided biopsy is preferable before definitive intervention [11,12,13]. Imaging therefore changes not only what is seen, but what is done next: operate, redirect to another local modality, continue systemic therapy, obtain tissue, or reassess.

Cervical cancer provides a concrete example of decision-oriented longitudinal MRI. Before treatment, T2-weighted MRI defines tumor size and local extension relevant to multidisciplinary selection between radical surgery and definitive chemoradiotherapy [12]. During and after chemoradiotherapy, serial MRI can quantify changes in tumor diameter or volume and characterize residual tissue; diffusion-weighted imaging can improve the assessment of residual tumor, whereas absolute or delta apparent diffusion coefficient values should not be used as universal response thresholds because acquisition and measurement remain insufficiently standardized [14]. The clinically useful quantitative metric is therefore not merely a number, but a reproducible measurement linked to a treatment decision.

A specific segmentation-to-plan example is MRI-guided brachytherapy for cervical cancer. Segmentation of the residual gross tumor volume at brachytherapy (GTV-Tres), high-risk clinical target volume (HR-CTV), and organs at risk helps determine whether an intracavitary applicator provides adequate coverage or whether an interstitial component is required. These contours are used to optimize the dose covering 90% of the HR-CTV (D90) while constraining the dose to the most exposed 2 cm^3^ (D2 cm^3^) of the bladder, rectum, sigmoid, and bowel [12,15]. Under-segmentation of residual parametrial disease may result in geographic miss, whereas inclusion of treatment-related fibrosis may unnecessarily enlarge the target and increase organ-at-risk exposure. The value of improved segmentation should therefore be assessed not only by contour-overlap metrics, but also by its effect on applicator choice, target coverage, organ-at-risk dose, and treatment delivery.

Building on previously described windows of surgical opportunity in gynecologic oncology [1], we operationalize the disease-modifying surgical window as open, uncertain, or closed. It is open when six proposed decision domains align: (1) a clearly defined and clinically meaningful target; (2) no competing disease that would negate the expected value of local treatment; (3) a disease trajectory compatible with spatially limited persistence or progression rather than generalized treatment failure; (4) technically achievable local control with acceptable anticipated morbidity; (5) a context-specific and clinically plausible advantage of surgery over biopsy, radiotherapy, image-guided ablation, systemic treatment, or observation; and (6) patient fitness and timing compatible with intervention. Technical resectability alone is therefore insufficient to define an open disease-modifying surgical window.

The window is uncertain when one or more decision-critical domains remain unresolved but could reasonably be clarified through additional imaging, short-interval reassessment, biopsy, or observation of response to systemic treatment. It is closed when one or more decisive domains are absent, particularly because of competing dissemination, generalized or rapidly multifocal progression, inability to achieve meaningful local control, disproportionate morbidity, or lack of a distinct advantage over nonsurgical management. These categories describe the current proportional value of disease-modifying surgery rather than permanent operability. They are multidisciplinary, time-dependent, and revisable, and should not be determined by imaging alone. Closure of the disease-modifying surgical window does not exclude separately justified diagnostic, salvage, symptom-directed, or palliative surgery.

### Illustrative Clinical Scenarios

The following hypothetical scenarios illustrate the proposed framework and are not intended as validated clinical criteria.

**Open window with imaging-target mismatch.** Following systemic therapy, a radiologically dominant pelvic mass decreases in size, while a smaller para-aortic lesion enlarges and no other disease progresses. The smaller lesion, rather than the dominant residual mass, represents the clinically relevant target because its behavior suggests spatially restricted treatment resistance. If complete local control is technically achievable with acceptable morbidity and may offer a context-specific advantage over biopsy, radiotherapy, or continued systemic treatment, imaging supports an open disease-modifying surgical window.

**Uncertain window.** A technically resectable pelvic recurrence is accompanied by newly detected, indeterminate peritoneal nodules. Although the principal lesion could be removed, the potential presence of occult dissemination makes the disease-modifying value of surgery uncertain. Additional imaging, short-interval reassessment, or biopsy should therefore precede definitive local treatment.

**Closed window.** A locally resectable pelvic lesion is identified during rapidly evolving multifocal progression at distant or peritoneal sites. Removal of the visible lesion is unlikely to alter the overall disease trajectory and is unlikely to offer a distinct advantage over systemic or nonsurgical local treatment. The disease-modifying surgical window is therefore closed, although separately justified diagnostic, salvage, symptom-directed, or palliative surgery may remain appropriate.

Imaging immediately before intervention should confirm that the target and therapeutic context remain unchanged, especially when substantial time has elapsed since the examination on which the decision was based. Post-intervention imaging should be interpreted against the predefined objective, such as complete local control, removal of the progressing site, or prevention of anticipated local complications, rather than as routine surveillance alone.

**Figure 1:**
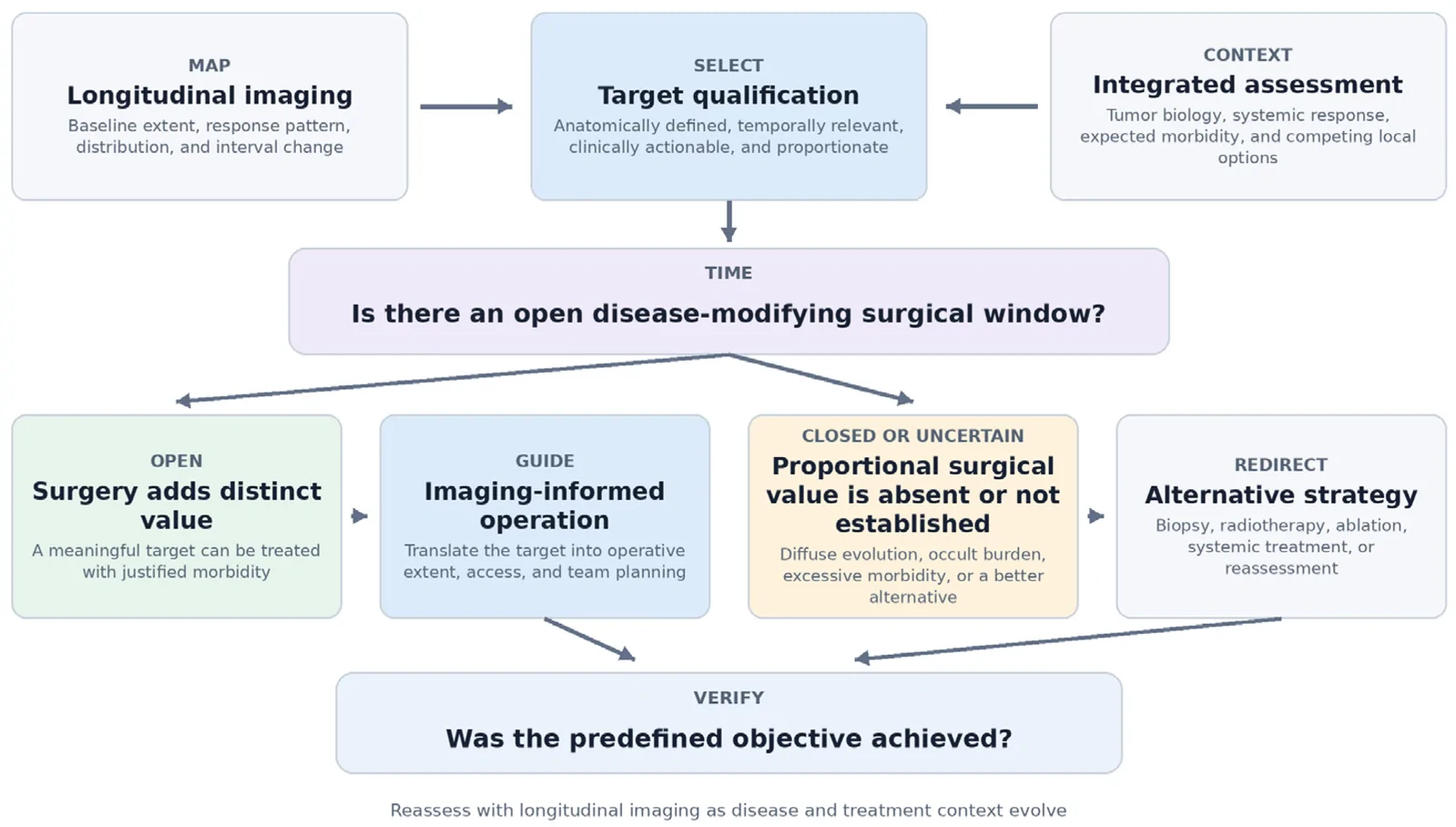
**The imaging-defined surgical decision.** Imaging functions as a longitudinal decision system that maps disease evolution, qualifies the clinically meaningful target, defines whether a surgical window is open, guides proportionate intervention, and verifies achievement of the predefined objective. When surgery lacks distinct value, imaging redirects management toward biopsy, another local modality, systemic treatment, or reassessment.

Imaging should therefore accompany the surgical decision throughout the disease course: baseline mapping, response reassessment, target confirmation, procedural guidance, and verification of local control. Its value lies not only in showing disease, but in demonstrating when the relationship between disease extent, treatment response, and surgical purpose has materially changed. This framework is summarized in Figure 1.

The decision consequences of common longitudinal imaging states are summarized in Table 1.

Although the proposed framework is intentionally cross-disease, it does not create interchangeable indications for cytoreduction, salvage surgery, or other local treatment. In ovarian, tubal, and primary peritoneal cancer, imaging primarily supports selection for primary, interval, or selected secondary cytoreduction, for which the probability of complete gross resection and the systemic-treatment context are central [2,3,4,16,17]. In cervical cancer, imaging guides primary selection between surgery and chemoradiotherapy and may support salvage surgery only in carefully selected localized recurrence when complete resection is plausible and competing pelvic or distant disease has been excluded [2,12,13]. In endometrial cancer, imaging defines uterine extent and the anatomical pattern of recurrence; decisions among resection, radiotherapy, and systemic treatment additionally depend on prior treatment and histopathological and molecular risk [2,18]. The shared concepts of target, trajectory, competing disease, anatomical feasibility, and uncertainty should therefore be applied within each disease-specific evidence base rather than transferred as uniform surgical criteria (Table 2).

Randomized evidence in platinum-sensitive recurrent ovarian cancer illustrates why anatomical resectability should not be equated with surgical benefit. In DESKTOP III, patients selected using a positive AGO score achieved complete resection in 75.5% of cases, and secondary cytoreduction improved median overall survival from 46.0 to 53.7 months (hazard ratio 0.75). By contrast, GOG-0213 used investigator-determined resectability; complete gross resection was achieved in 67% of operated patients, 84% received bevacizumab, and surgery did not improve overall survival (50.6 versus 64.7 months; hazard ratio 1.29) [16,17]. These findings do not establish the superiority of one selection strategy because the trial populations, systemic treatment, and study designs differed. Rather, they demonstrate that limited-volume, apparently resectable recurrence does not by itself define an open disease-modifying surgical window. Imaging must be integrated with formal clinical selection, the likelihood of complete resection, systemic treatment context, disease trajectory, and patient fitness; surgical benefit cannot be inferred from imaging alone.

**Table 2:** Disease-specific translation and boundaries of the decision-oriented imaging framework.

Disease Context	Decision-Oriented Imaging Role	Principal Pitfall and Future Need
Ovarian, tubal, and primary peritoneal cancer	Imaging supports selection between primary cytoreduction and systemic treatment. CT and diffusion-weighted MRI map peritoneal, mesenteric, and small-bowel disease and may identify response-selected targets for interval or recurrent local treatment [2,3,4].	Small serosal or mesenteric implants and treatment-related changes may remain occult. Standardized lesion matching and decision-linked resectability reporting require prospective validation.
Cervical cancer	Expert pelvic ultrasound (transvaginal/transrectal, with transabdominal assessment as appropriate) or MRI defines tumor size and local extension for treatment selection. Both may flag suspicious pelvic nodes but cannot exclude small-volume metastases; CT or FDG PET/CT assesses nodal and distant disease according to stage and risk. Serial MRI supports response assessment and brachytherapy planning [2,12,14,15].	Ultrasound is operator- and expertise-dependent, and negative ultrasound or MRI cannot exclude small-volume nodal metastases. Fibrosis and inflammation may mimic residual tumor; ADC thresholds and segmentation methods require harmonization.
Endometrial cancer	Expert pelvic ultrasound (transvaginal/transrectal and transabdominal, as appropriate) or pelvic MRI assesses tumor size, myometrial invasion, and cervical stromal invasion. These findings guide fertility-sparing or tailored surgery. Ultrasound may flag suspicious accessible nodes; risk-adapted CT or FDG PET/CT provides comprehensive nodal, peritoneal, and distant assessment [2,18].	Performance is operator- and reader-dependent, and ultrasound, MRI, CT, or FDG PET/CT may miss small-volume nodal disease. Imaging should be integrated with histopathological and molecular risk rather than used as an independent treatment selector.
Vulvar and vaginal cancer	Ultrasound and MRI map local extension relevant to conservative versus extended surgery. Nodal ultrasound with biopsy and CT or FDG PET/CT can influence groin, pelvic, chemoradiotherapy, or exenterative strategies [2,19,20].	Superficial disease, nodal micrometastases, and post-treatment change remain difficult to assess. Standardized local and nodal reporting requires multicenter validation.

## 5. Imaging Target Mismatch

The lesion that is easiest to detect is not always the lesion that should be treated. Conventional imaging naturally directs attention toward the largest, most metabolically active, or most anatomically conspicuous abnormality. Surgical relevance, however, depends on a different set of considerations: whether the lesion represents active disease, whether it is responsible for treatment failure or impending morbidity, and whether its local control is likely to alter subsequent management.

For operational purposes, imaging-target mismatch is present when the lesion that is radiologically dominant by size, conspicuity, or metabolic activity is not the lesion that ranks highest for local intervention after consideration of five decision domains: lesion-level trajectory, likelihood of clinically consequential progression, value for tissue acquisition, anatomical feasibility, and expected therapeutic benefit. The mismatch is clinically relevant only when this discordance could change whether local intervention is justified, which lesion should be treated or sampled, or which local modality is preferred. It should therefore prompt explicit multidisciplinary comparison of candidate targets and competing disease rather than automatic selection of the largest, most avid, or most accessible lesion. Imaging-target mismatch is a proposed decision-support construct, not a validated diagnostic category, scoring system, or independent indication for surgery. Several forms of mismatch may occur:

- The largest lesion is responding, while a smaller lesion is progressing;
- The most metabolically active lesion is not safely resectable;
- The most accessible lesion is suitable for biopsy but not relevant to disease control;
- The lesion most likely to contain resistant tumor is not the lesion routinely selected for measurement;
- Apparently isolated disease is accompanied by occult dissemination that makes major local intervention disproportionate.

Traditional response criteria do not resolve this problem because they were designed to standardize evaluation rather than identify the optimal local target [5]. The distinction is especially important in mixed response, when lesions follow different trajectories during the same systemic therapy. A patient-level label of partial response or stable disease may then obscure the site driving the next decision.

Imaging-target mismatch should prompt multidisciplinary reassessment rather than automatic selection of the most visible lesion. Serial anatomical change, metabolic behavior, clinical consequences, procedural accessibility, and the expected value of tissue should be considered together. When uncertainty remains, image-guided biopsy may determine whether the proposed target contains viable or biologically distinct tumor without committing the patient to a larger operation [11].

The purpose of identifying mismatch is not to infer tumor biology from imaging. It is to expose uncertainty and prevent radiological conspicuity from being mistaken for therapeutic importance. A legitimate target is the lesion whose local treatment is most likely to alter the clinical course, not necessarily the most prominent lesion on a single examination.

## 6. From Preoperative Mapping to Intraoperative Guidance

Preoperative imaging translates disease distribution into an operative strategy. It maps lesion number and location, vascular relationships, organ involvement, and the potential need for multidisciplinary surgery. Structured review can also identify sites where complete resection is unlikely or morbidity may be excessive. Conventional two-dimensional review may be inadequate in complex recurrence, particularly after surgery or radiotherapy has distorted normal tissue planes [13].

Three-dimensional segmentation and patient-specific reconstruction may convert cross-sectional imaging into an interactive surgical map. In a prospective proof-of-concept study of four patients undergoing surgery for complex gynecologic cancer recurrence, static digital twins supported anatomical review, operative planning, and multidisciplinary communication [6]. The study demonstrates feasibility but is too small to establish improved oncologic outcomes or support routine use.

The next step is registration of preoperative imaging with the operative field. Intraoperative ultrasound, fluorescence imaging, and digital anatomical models can assist lesion localization, vascular assessment, lymphatic mapping, and detection of disease that is difficult to distinguish by inspection or palpation alone [21]. These technologies address different questions and should not be treated as interchangeable.

Tumor-targeted fluorescence provides a relevant example. In a phase III study of folate receptor-positive ovarian cancer, pafolacianine identified additional malignant lesions that had not been planned for resection using conventional surgical evaluation [22]. This demonstrates that intraoperative imaging can modify the operative map in real time. It does not, however, establish that every additionally detected lesion should be removed or that increased lesion detection necessarily improves survival.

Intraoperative guidance should therefore remain linked to a predefined surgical objective. Technologies that reveal additional disease are valuable only when the resulting action is clinically justified and does not lead to unplanned, disproportionate extension of surgery. The aim is not maximal visualization for its own sake, but better alignment between the preoperative target, the operative anatomy, and the intended therapeutic result.

The transition from mapping to guidance can therefore be summarized as preoperative localization, operative translation, real-time confirmation, and proportionate modification of the surgical plan.

## 7. When Imaging Should Close the Surgical Window

Imaging can support surgery, but it should also identify when an operation is unlikely to achieve its intended purpose. Technical resectability of one visible lesion is insufficient if the overall disease distribution indicates that local intervention will not meaningfully alter the therapeutic trajectory.

In this framework, closure refers specifically to the current disease-modifying surgical window. It means that the proposed operation no longer has a plausible and proportionate capacity to alter disease trajectory, even when an individual lesion remains technically resectable. It does not exclude a separately justified diagnostic, salvage, symptom-directed, or palliative procedure [12,13].

The disease-modifying window may be closed when imaging shows widespread progression, multiple new lesions, extensive small-bowel or mesenteric involvement, or disease at critical anatomical sites where complete local control would require disproportionate morbidity. Rapid progression between examinations may also indicate that an apparently confined target is only one manifestation of evolving systemic disease. In this setting, removing the visible lesion is unlikely to address the process driving treatment failure.

Discordance between the intended surgical objective and the imaging findings is another warning sign. Resection of a presumed resistant focus is difficult to justify when other sites are progressing simultaneously. Consolidative treatment for oligopersistent disease is less plausible when residual sites are widely distributed or cannot all be treated safely. Surgery solely for tissue acquisition may also be disproportionate when image-guided biopsy can provide adequate material with substantially lower morbidity [11].

Imaging should also support comparison with nonsurgical options. Depending on target size, location, access, proximity to critical structures, anticipated morbidity, and the need to avoid interrupting systemic therapy, stereotactic radiotherapy or image-guided ablation may be preferable to surgery [12,13]. Conversely, unresolved imaging uncertainty may justify short-interval reassessment or tissue confirmation before definitive local treatment.

Closure of the surgical window is not a permanent declaration of unresectability. A later systemic response may reduce disease burden or create a new opportunity for localized treatment. Imaging therefore provides a time-dependent assessment that should be revised as disease trajectory and treatment context change.

## 8. Implications for Radiological Reporting and Multidisciplinary Practice

For imaging to function as a surgical decision system, the radiology report must do more than list disease sites or assign response. When the referral question concerns local intervention, reporting should make the decision logic visible. Six principles can provide a practical structure [23].

**1.**  **Define the clinical decision and candidate target.** The report should identify the intervention being considered and distinguish the radiologically dominant or routinely measured lesion from the lesion that is clinically actionable. If several targets are plausible, they should be ranked according to therapeutic relevance rather than size or conspicuity alone. The conclusion should state whether a coherent local target is present and what objective its treatment might serve.
**2.**  **Describe lesion-level trajectory.** Comparison should extend beyond total disease burden or a patient-level response category. Each candidate target should be assessed against relevant prior examinations, including the direction and rate of change, behavior during therapy, and concordance with disease elsewhere. Mixed response, oligopersistence, and oligoprogression should be stated explicitly, including whether progression is confined to one or a few sites while the remaining disease is controlled [5,7].
**3.**  **Report anatomy for the competing local options.** For surgery, the report should describe the anatomical compartment, depth and extent of organ involvement, relationships to vessels, bowel and mesentery, urinary tract, pelvic sidewall, nerves, and bone, and the effects of previous surgery or radiotherapy. It should also identify features that alter the feasibility of ablation or stereotactic radiotherapy, including percutaneous access, lesion motion, proximity to thermally or radiation-sensitive structures, and whether all active sites can be covered [9,10]. This allows the multidisciplinary team to compare modalities on the same anatomical basis [24,25].
**4.**  **Search for competing disease and state the limits of exclusion.** Any peritoneal, serosal, mesenteric, nodal, or distant abnormality that could invalidate a local strategy should be described, even when equivocal. The report should distinguish no visible competing disease from confident exclusion of dissemination, which may not be possible for small-volume or diffuse disease [2,3,4]. When the examination is technically limited, the limitation and its likely impact on the decision should be explicit.
**5.**  **Convert uncertainty into a next step.** Findings should be categorized as definite, probable, or indeterminate when uncertainty affects the decision, with a brief explanation of its source. Short-interval reassessment is appropriate when temporal evolution is likely to resolve uncertainty without compromising outcome; image-guided biopsy should be suggested when tissue confirmation could change management and a lower-morbidity route is available [11]. Additional imaging should be recommended only when it is likely to answer a specific decision-limiting question.
**6.**  **Provide a decision-oriented impression.** The impression should integrate five elements: target, trajectory, competing disease, anatomical feasibility, and residual uncertainty. It may state whether imaging supports an open, uncertain, or closed disease-modifying surgical window, while avoiding an imaging-only declaration of operability. A brief statement of the principal reason, such as isolated progression, unconfirmed dissemination, or disproportionate anatomical risk, makes the report directly usable in multidisciplinary discussion [23,25].

## 9. Limitations of Imaging-Based Decision-Making

No imaging modality can determine the value of surgery in isolation. Reader agreement depends on the clinical question, acquisition protocol, reader experience, disease distribution, and treatment-related anatomical change. Variability is particularly relevant when assessing subtle peritoneal, serosal, or mesenteric disease, mixed response, and the distinction between viable tumor and fibrosis or inflammation. In one prospective comparison, interobserver agreement for predicting incomplete resection was almost perfect for whole-body diffusion-weighted MRI but only moderate for CT, illustrating that reproducibility is both task- and modality-specific [26]. Structured reporting, consistent lesion matching, access to previous examinations, and review by an experienced multidisciplinary team may reduce, but cannot eliminate, this uncertainty [4,23,24,25]. Disagreement or indeterminate findings should therefore be reported explicitly rather than converted into a binary declaration of operability.

Each modality also has specific blind spots. CT is widely available and provides an effective overview of disease, but may underestimate subcentimeter or isodense peritoneal implants and involvement of the bowel serosa or mesentery. Diffusion-weighted MRI can improve peritoneal mapping, but its performance depends on optimized acquisition, artifact control, and reader expertise. FDG PET/CT may reveal otherwise occult extraperitoneal disease but can miss small or hypometabolic lesions and cannot exclude limited peritoneal dissemination. Ultrasound provides high-resolution pelvic assessment but remains operator- and access-dependent and does not replace whole-body mapping [2,3,4]. Post-treatment inflammation, fibrosis, and anatomical distortion may further mimic or obscure viable disease across modalities. A negative examination should therefore be expressed as showing no visible competing disease within the limits of the technique, rather than as excluding occult dissemination. When residual uncertainty could change management, additional imaging, short-interval reassessment, biopsy, or surgical assessment should be considered.

The targeted and non-systematic selection of literature may have introduced selection bias and precludes any claim of exhaustive evidence coverage.

## 10. Validation and Research Priorities

The clinical value of an imaging marker cannot be established merely by showing an association with prognosis, treatment response, or the probability of complete resection. A marker may identify patients with inherently favorable disease without demonstrating that they derive greater benefit from surgery than from a nonsurgical strategy. The relevant question is comparative: does the imaging finding modify the incremental value of surgery relative to an appropriate alternative [27]?

### 10.1. A Staged Validation Pathway

Validation of the proposed constructs should proceed in stages. First, a standardized reporting template should translate each construct into observable decision domains. In prospective imaging datasets, radiologists should independently record the candidate target, lesion-level trajectory, competing disease, anatomical feasibility, and residual uncertainty. Multidisciplinary panels should then integrate these findings with treatment response, patient fitness, anticipated morbidity, and alternative local options to classify the disease-modifying surgical window as open, uncertain, or closed. Imaging-target mismatch should be recorded when the radiologically dominant lesion differs from the highest-ranked target for local intervention. Agreement, unclassifiable cases, and reasons for discordance should be reported, with multidisciplinary consensus treated as a clinical comparator rather than an infallible reference standard [23,27].

Second, prospective multicenter observational studies should record the framework-based assessment before multidisciplinary discussion and determine whether it changes target selection, treatment modality, the need for biopsy or short-interval reassessment, or the decision to operate. Classification should subsequently be compared with management decisions, operative findings, pathology, morbidity, local control, and the early disease course. Third, clinical utility should be evaluated by comparing decision-oriented reporting with usual practice, preferably using cluster-randomized or stepped-wedge designs. The framework should be considered clinically useful only if it reduces non-therapeutic operations or avoidable morbidity, improves treatment selection or timing, or permits continuation of the current systemic regimen when clinically justified without compromising oncological outcomes. External validation across institutions, readers, imaging platforms, and disease settings would be required before routine implementation.

Relevant endpoints should extend beyond radiological accuracy. They may include:

Avoidance of non-therapeutic or incomplete operations;
Achievement of the predefined local-control objective;
Change in surgical extent or multidisciplinary team composition;
Treatment-related morbidity;
Generation of management-changing tissue;
Continuation of the current systemic treatment when clinically justified;
Time to the next systemic-treatment change;
Quality of life and patient-reported outcomes.

Radiomics and artificial intelligence models require particular caution. Many published models are retrospective, developed on restricted single-center datasets, and evaluated using internal discrimination metrics rather than clinical utility. Apparent performance may not persist when patient characteristics, disease prevalence, scanner vendor, acquisition protocol, reconstruction method, annotation practice, or clinical workflow changes. This dataset shift may be compounded by underspecification, whereby models with similar internal accuracy rely on different and potentially non-robust features and consequently behave differently after deployment. Multicenter external validation should therefore be complemented by stress testing, subgroup-specific performance analysis, calibration assessment, prospective silent deployment, and continuous post-implementation monitoring [28,29,30].

Explainability can support error analysis and clinician oversight, but a heat map or feature-attribution output does not establish causal validity and may itself be unstable. Training datasets should be audited for representation and labeling bias, with clinically relevant differences in performance across demographic and disease subgroups reported explicitly. Generative systems used for report drafting or decision support introduce the additional risk of plausible but unsupported outputs (“hallucinations”) and therefore require source grounding and mandatory human verification. Ethical and legal implementation must also address data governance, privacy, cybersecurity, automation bias, accountability, and the allocation of responsibility when algorithmic advice contributes to harm [31,32]. Within the proposed framework, AI may quantify lesion trajectories, flag potential imaging-target mismatch, or identify cases requiring reassessment; however, it should not autonomously define the surgical target or declare the surgical window open or closed. These remain multidisciplinary clinical judgments. AI should therefore function as a decision-support tool that makes evidence and uncertainty more explicit, rather than as a substitute for radiological or surgical judgment [30,31,32].

### 10.2. Emerging Imaging Biomarkers and Treatment-Planning Techniques

Beyond anatomical mapping, functional and molecular imaging may provide candidate biomarkers of tumor cellularity, metabolism, receptor expression, or stromal activity. Diffusion-derived MRI measurements may contribute to response assessment, while non-FDG PET tracers can provide phenotype-specific information. 68Ga-FAPI PET/CT has demonstrated high lesion-to-background contrast in small, heterogeneous gynecologic cancer cohorts, and 18F-FES PET has shown associations with estrogen-receptor biology and prognosis in endometrial carcinoma; neither, however, has an established role in routine surgical or radiotherapy selection [14,33,34]. For treatment planning, high-resolution multiplanar T2-weighted MRI remains the anatomical backbone, complemented by diffusion-weighted imaging for residual-tumor conspicuity, contrast-enhanced sequences when viable tissue remains uncertain, and three-dimensional reconstruction or segmentation for applicator selection, target and organ-at-risk contouring, dose optimization, or complex surgical mapping [6,15,35]. Reports incorporating quantitative or molecular biomarkers should specify the acquisition, segmentation, comparison method, and residual uncertainty. These approaches should ultimately be validated by whether they change target definition, modality selection, operative extent, or delivered dose, rather than by image quality or association with outcome alone.

Modality selection should begin with the decision-limiting clinical question. Expert pelvic ultrasound is appropriate for high-resolution dynamic assessment of a cervical or endometrial primary, adnexal disease, or an accessible nodal or biopsy target, but it cannot exclude disease outside the field of examination [2,3,18,19,20]. MRI is most useful when decisions depend on delineation of local tissue planes; stromal, parametrial, pelvic sidewall, or adjacent-organ involvement; post-treatment response; or brachytherapy and salvage planning. Its interpretation remains dependent on protocol quality and reader expertise, and fibrosis or inflammation may mimic viable tumor [4,14,15,26,35]. Contrast-enhanced CT provides rapid thoraco-abdominopelvic mapping when peritoneal, mesenteric, bowel, vascular, nodal, or distant disease may alter the feasibility or proportional value of local treatment, although small-volume serosal or infiltrative disease may remain occult [2,4,24,25]. FDG PET/CT is most useful when otherwise occult nodal or distant disease, or metabolic characterization of a candidate target, could redirect management; inflammatory uptake may reduce specificity, and a negative examination does not exclude small or low-avidity disease [2,12,24]. Additional imaging should therefore be recommended when it can resolve a specific uncertainty that would change target selection, treatment modality, or timing. The complementary strengths, limitations, and decision-oriented applications of these imaging modalities are summarized in Table 3.

**Table 3:** Complementary strengths and decision-oriented applications of principal imaging modalities.

Imaging Modality	Principal Strength	Representative Decision-Oriented Application	Principal Limitation
Expert pelvic ultrasound	Dynamic, high-resolution assessment of pelvic organs and accessible nodes	Defines local cervical or endometrial tumor extent, characterizes adnexal disease, and guides biopsy of accessible targets [2,3,18,19,20]	Operator- and access-dependent; does not provide comprehensive whole-body mapping
Contrast-enhanced CT	Rapid and widely available thoraco-abdominopelvic mapping	Demonstrates peritoneal, mesenteric, bowel, vascular, nodal, or distant disease that may alter the feasibility or proportional value of surgery [2,4,24,25]	May underestimate small-volume peritoneal or infiltrative disease
MRI, including DWI and contrast-enhanced sequences	Superior soft-tissue contrast and local anatomical characterization	Assesses stromal, parametrial, myometrial, pelvic sidewall, or adjacent-organ involvement and supports response assessment and brachytherapy planning [4,14,15,26,35]	Performance depends on protocol quality and reader expertise; fibrosis and inflammation may mimic residual tumor
FDG PET/CT	Whole-body metabolic assessment	Detects otherwise occult competing disease or identifies an isolated metabolically active lesion that may represent treatment-resistant tumor [2,12,24]	May miss small or low-avidity lesions; inflammatory uptake may reduce specificity
Novel molecular PET tracers	Phenotype-, receptor-, or stroma-specific characterization	FAPI- or FES-based imaging may support biologically informed target selection or biopsy in selected research settings [33,34]	Evidence remains preliminary, and routine treatment-selection roles are not established
Segmentation and image-guided or intraoperative techniques	Translation of imaging information into procedural anatomy	Supports target contouring, applicator selection, biopsy or ablation access, intraoperative localization, and modification of operative extent [6,11,15,21,22,35]	Clinical value depends on a predefined therapeutic objective and remains incompletely validated

The strongest validation would come from prospective studies comparing framework-based reporting with usual multidisciplinary practice. Evaluation should focus on whether the framework improves target and treatment selection, reduces non-therapeutic operations or avoidable morbidity, and improves patient outcomes.

## 11. Conclusions

Imaging in gynecologic oncology should no longer be viewed solely as a tool for diagnosis, staging, and static assessment of resectability. Its contemporary role is longitudinal: to map disease evolution, identify a clinically meaningful target, determine whether the disease-modifying surgical window is open, uncertain, or closed, guide the intervention, and verify whether the intended objective has been achieved.

An imaging-defined target is not merely a visible or technically resectable lesion. It is a lesion for which local treatment could credibly alter the therapeutic course and for which surgery may offer a context-specific and clinically plausible advantage over biopsy, radiotherapy, ablation, systemic therapy, observation, or another local option. Identifying imaging-target mismatch helps prevent radiological prominence from being mistaken for surgical relevance.

Quantitative imaging, three-dimensional reconstruction, intraoperative molecular imaging, radiomics, and artificial intelligence may improve target definition and treatment planning, but their clinical utility requires decision-linked and externally validated evidence. Imaging should support, not replace, multidisciplinary judgment. Future radiology reports should move beyond disease burden and apparent resectability to communicate the target, lesion-level trajectory, competing disease, residual uncertainty, and the relevance of each finding to the proposed decision.

## Figures and Tables

**Table 1:** Author-proposed, unvalidated conceptual framework for decision-oriented imaging across common surgical decision states.

Clinical State	Key Imaging Question	Findings That May Support Surgery	Findings That May Argue Against Surgery
Primary or interval surgery	Can a meaningful complete resection be achieved?	Anatomically controllable disease with a defined therapeutic objective.	Diffuse mesenteric or small-bowel disease, competing dissemination, or morbidity disproportionate to the objective.
Major systemic response	Has treatment opened a surgical window?	Marked global response with a limited residual target whose local control could alter the treatment trajectory.	Persistent widespread active disease or rapid multifocal evolution despite response at selected sites.
Oligopersistence	Is residual disease finite and locally controllable?	All clinically relevant residual sites can be treated with a coherent local-control objective.	Multiple competing untreated sites or residual disease that cannot be comprehensively controlled.
Oligoprogression	Is this truly spatially restricted treatment escape?	One or a few progressing lesions while the remaining disease remains controlled by systemic therapy.	Simultaneous progression at multiple sites or evidence that the apparent target is only one manifestation of systemic escape.
Suspected resistant focus	Is tissue needed, and would local control add value?	Resection could provide both meaningful local control and management-changing tissue.	Image-guided biopsy can answer the tissue question with lower morbidity and no separate therapeutic reason for resection.
Complex recurrence	Is surgery proportionate to the expected benefit?	A clearly defined target can be removed with acceptable morbidity and a plausible disease-modifying or salvage objective.	Critical anatomical involvement or expected morbidity is disproportionate to the likely change in therapeutic trajectory.
Immediate preoperative reassessment	Is the original surgical decision still valid?	The target, disease distribution, and intended objective remain unchanged and no competing disease has emerged.	New lesions, rapid interval progression, newly apparent dissemination, or loss of the original therapeutic rationale.

Note. This author-proposed framework is unvalidated. The findings are decision prompts rather than established imaging-only criteria for operability; final management remains disease-specific, multidisciplinary, and context-dependent.

## Data Availability

No new data were created or analyzed in this study. Data sharing is not applicable to this article.
